# Risk factors associated with the epilepsy treatment gap in Kilifi, Kenya: a cross-sectional study

**DOI:** 10.1016/S1474-4422(12)70155-2

**Published:** 2012-08

**Authors:** Caroline K Mbuba, Anthony K Ngugi, Greg Fegan, Fredrick Ibinda, Simon N Muchohi, Christopher Nyundo, Rachael Odhiambo, Tansy Edwards, Peter Odermatt, Julie A Carter, Charles R Newton

**Affiliations:** a KEMRI/Wellcome Trust Research Programme, Centre for Geographic Medicine Research (Coast), Kilifi, Kenya; b Department of Infectious Disease Epidemiology, Faculty of Epidemiology and Population Health, London School of Hygiene and Tropical Medicine, London, UK; c Tropical Epidemiology Group, London School of Hygiene and Tropical Medicine, London, UK; d Clinical Research Unit, London School of Hygiene and Tropical Medicine, London, UK; e Studies of Epidemiology of Epilepsy in Demographic Surveillance Systems (SEEDS), International Network for Demographic Evaluation of Populations and their Health (INDEPTH), Accra, Ghana; f Department of Public Health and Epidemiology, Swiss Tropical and Public Health Institute, Basel, Switzerland; g University of Basel, Basel, Switzerland; h Centre for International Health and Development, Institute of Child Health, University College London, London, UK; i Neurosciences Unit, Institute of Child Health, University College London, London, UK; j Centre for Clinical Vaccinology and Tropical Medicine, Nuffield Department of Clinical Medicine, University of Oxford, Oxford, UK; k Department of Psychiatry, University of Oxford, Oxford, UK

## Abstract

**Background:**

Many people with epilepsy in low-income countries do not receive appropriate biomedical treatment. This epilepsy treatment gap might be caused by patients not seeking biomedical treatment or not adhering to prescribed antiepileptic drugs (AEDs). We measured the prevalence of and investigated risk factors for the epilepsy treatment gap in rural Kenya.

**Methods:**

All people with active convulsive epilepsy identified during a cross-sectional survey of 232 176 people in Kilifi were approached. The epilepsy treatment gap was defined as the percentage of people with active epilepsy who had not accessed biomedical services or who were not on treatment or were on inadequate treatment. Information about risk factors was obtained through a questionnaire-based interview of sociodemographic characteristics, socioeconomic status, access to health facilities, seizures, stigma, and beliefs and attitudes about epilepsy. The factors associated with people not seeking biomedical treatment and not adhering to AEDs were investigated separately, adjusted for age.

**Findings:**

673 people with epilepsy were interviewed, of whom 499 (74%) reported seeking treatment from a health facility. Blood samples were taken from 502 (75%) people, of whom 132 (26%) reported taking AEDs, but 189 (38%) had AEDs detectable in the blood. The sensitivity and specificity of self-reported adherence compared with AEDs detected in blood were 38·1% (95% CI 31·1–45·4) and 80·8% (76·0–85·0). The epilepsy treatment gap was 62·4% (58·1–66·6). In multivariable analysis, failure to seek biomedical treatment was associated with a patient holding traditional animistic religious beliefs (adjusted odds ratio 1·85, 95% CI 1·11–2·71), reporting negative attitudes about biomedical treatment (0·86, 0·78–0·95), living more than 30 km from health facilities (3·89, 1·77–8·51), paying for AEDs (2·99, 1·82–4·92), having learning difficulties (2·30, 1·29–4·11), having had epilepsy for longer than 10 years (4·60, 2·07–10·23), and having focal seizures (2·28, 1·50–3·47). Reduced adherence was associated with negative attitudes about epilepsy (1·10, 1·03–1·18) and taking of AEDs for longer than 5 years (3·78, 1·79–7·98).

**Interpretation:**

The sensitivity and specificity of self-reported adherence is poor, but on the basis of AED detection in blood almost two-thirds of patients with epilepsy were not on treatment. Education about epilepsy and making AEDs freely available in health facilities near people with epilepsy should be investigated as potential ways to reduce the epilepsy treatment gap.

**Funding:**

Wellcome Trust.

## Introduction

More than 62 million people with epilepsy live in countries with low and middle incomes^1^ and most of them do not receive appropriate treatment, an issue known as the epilepsy treatment gap.^2^ The epilepsy treatment gap is defined as the number of people with active epilepsy who have not accessed biomedical services (ie, epilepsy not diagnosed) or who are not on treatment or are on inadequate treatment, expressed as a percentage of the total number with active epilepsy.^2^ In a systematic review of studies done in countries with low and middle incomes, the overall estimate of the epilepsy treatment gap based on self-reporting was 56%, but the 95% CI was wide, ranging from 31% to 100%, with a higher proportion in rural areas than in urban areas.^3^ A more recent review reported an epilepsy treatment gap of more than 75% in low-income countries compared with less than 10% in high-income countries.^4^

The epilepsy treatment gap could be caused by poor access to diagnosis and treatment of epilepsy or non-adherence to antiepileptic drugs (AEDs). Adherence to a drug regimen is defined as the extent to which patients take drugs as prescribed by their health-care providers.^5^ Unintentional non-adherence might be due to forgetfulness or inability to follow treatment instructions because of poor understanding or impairment (eg, poor eyesight), whereas intentional non-adherence arises when the patient rejects either the doctor's diagnosis or recommended treatment. Between 25% and 75% of people with epilepsy do not follow prescribed drug regimens, leading to uncontrolled seizures and reduced quality of life.^6^

Health-care seeking behaviour and adherence are complex issues affected by many factors. They can be explored with Andersen's behavioural model,^7^ which considers that health service use and adherence are a function of four categories: health-care system factors (eg, distance to health facilities); predisposing factors (eg, health beliefs); enabling factors (eg, transportation); and need, which refers to severity of illness and whether people judge their illness to be of sufficient magnitude to consult health services.

We determined the treatment gap, investigated the reliability of self-reporting of adherence, and examined factors associated with failure to seek biomedical treatment and non-adherence in people with epilepsy in a rural area of Kenya.

## Methods

### Participants

We undertook a cross-sectional survey and risk-factor analysis of the epilepsy treatment gap. The study was done in the Kilifi health and demographic surveillance system (KHDSS) with 233 881 residents in 2008.^8^ The residents are mainly Mijikenda, a Bantu group of nine tribes with Giriama (45%) dominating. The average per head income is about 700 Kenyan Shillings (KES; US$8) per month and about 55% of the population is regarded as poor. Most people (80%) depend on subsistence farming. Literacy is low (45%).^9^ KHDSS is served by one district hospital (Kilifi District Hospital, KDH), one health centre, and 12 dispensaries. KDH serves as a primary care centre and first-level referral facility for the district and stocks four AEDs: phenobarbital, phenytoin, carbamazepine, and sodium valproate. The health centres and dispensaries stock only phenobarbital.

People with epilepsy were identified in a three-stage cross-sectional survey that was done to establish the prevalence of active convulsive epilepsy as previously described.^10^ In the first stage, household heads were asked if anybody in the household had convulsions. People who were reported to have seizures were interviewed with an epilepsy-specific questionnaire^11^ and those who were classed as positive were invited to be assessed by a clinician. Active convulsive epilepsy was defined as two or more unprovoked tonic or clonic seizures in a lifetime, of which at least one seizure was within the 12 months preceding the survey, since this criterion is used for prescription of AEDs in Kenya,^12^ is used in other parts of Africa,^13^, ^14^ and is recommended by the International League Against Epilepsy for calculation of the treatment gap,^2^, ^15^ and there is poor documentation of previous seizures and recall beyond a year in this region. The study was reviewed and approved by the Kenya Medical Research Institute and national scientific and ethical review committees and the Swiss Tropical and Public Health Research Committee. All participants or their carers provided written informed consent.

### Procedures

Questionnaires based on those used in previous studies of epilepsy in this area^10^, ^16^, ^17^, ^18^, ^19^ and elsewhere^20^, ^21^ were developed in English and translated into local languages. In addition to the outcomes of not seeking treatment and non-adherence to AEDs, we obtained data for several other items: demographic details of the person with epilepsy (age and sex), social characteristics (religion,^22^ education, occupation, and marital status) of either the person with epilepsy or their carer if the carer made decisions about seeking treatment and taking AEDs if prescribed; socioeconomic data for the households based on assets;^23^ accessibility factors (distance from home to health facility, and payment for AEDs); severity of epilepsy (duration of epilepsy, seizure frequency, type of seizures, and injury during a seizure); stigma; and beliefs and attitudes about epilepsy. People with learning difficulties were defined as those who were not orientated for person, place, or the period of day (morning or afternoon) and who had difficulty following instructions—eg, finger nose test—without a neurological deficit as assessed by the clinician. For analysis of non-adherence, details of prescribed regimen were obtained (number of prescribed AEDs, frequency of taking AEDs per day, side-effects of AEDs, and number of years a person with epilepsy has taken AEDs). The field staff administered questionnaires to all people with a confirmed diagnosis of active convulsive epilepsy after written informed consent. If the person with epilepsy was a child or cognitively impaired adult, a caregiver was interviewed. For the risk factor analysis, people who sought biomedical treatment were compared with those who had not sought treatment, and those who adhered (assessed by presence of AEDs in blood) were compared with those who did not have AEDs detected in their blood.

Data were obtained from households for assets and indicators of household characteristics such as livestock and source of energy for cooking and lighting. Principal component analysis was done to construct a homestead wealth assets index from the range of assets and household characteristics.^24^ Households were classified into socioeconomic status quintiles on the basis of this index.^23^

Perceived stigma and epilepsy beliefs and attitudes were measured with Likert scales. The Kilifi stigma scale for epilepsy had 15 questions and measured only perceived stigma.^25^ The Kilifi epilepsy beliefs and attitude scale had 34 questions, divided into five subscales: causes of epilepsy, biomedical treatment of epilepsy, cultural treatment of epilepsy, risk and safety concerns, and negative stereotypes about epilepsy.^26^

To calculate distance to health facilities, homesteads and health facilities were mapped with a global positioning system and the Euclidean distance was calculated with the coordinates as a reflection of the time taken to travel the distance. The health facility used was defined as the one where people with epilepsy reported seeking treatment most frequently. Treatment-seeking behaviour was established by asking whether the person had ever sought treatment for epilepsy from a health facility before the 2008 epidemiological survey. People answering “yes” were asked to identify the health facility from which they sought treatment.

Questions were asked about the AEDs that patients were currently taking (actual tablets were shown on a board to aid recognition). Reported adherence was assessed with the Morisky medication adherence scale, a four-item scale with high reliability and validity^27^ that has been used to measure adherence in epilepsy.^28^, ^29^

To measure adherence, blood samples (2 mL) were collected. Phenobarbital was measured in all samples, with phenytoin and carbamazepine measured in those from participants taking these AEDs, with a TDx FLx analyser (Abbott Laboratories, Abbott Park, IL, USA). An individual was defined as adherent if AEDs were detected in their blood, with detectable limits of 1·1 μg/mL for phenobarbital, 1·0 μg/mL for phenytoin, and 0·5 μg/mL for carbamazepine. Since the half-lives of phenytoin and carbamazepine (the AEDs with the shortest elimination half-lives used in this area) are greater than 5 h,^30^ and it takes at least five half-lives to clear the drug after one dose, the absence of AED in the blood suggests that the drug has not been taken within at least the 24 h before the blood sample was taken. The optimum therapeutic ranges were defined as 10–40 μg/mL for phenobarbital, 10–20 μg/mL for phenytoin, and 4–12 μg/mL for carbamazepine.^31^

### Statistical analysis

Data were double-entered and verified with a MySQL V5 database. Statistical analyses were done with Stata versions 11 and 12. We investigated treatment seeking for all study participants interviewed, whereas non-adherence was investigated only in those who reported taking AEDs and gave a blood sample. The epilepsy treatment gap was defined as the number of people with active epilepsy who had not accessed biomedical services or who were not on treatment or were on inadequate treatment, expressed as a percentage of the total number with active epilepsy.^2^ The sensitivity, specificity, and confidence intervals were calculated in Stata. Concordance was assessed between self-reported adherence and AED detection in blood with the κ statistic. In exploratory analyses, we examined the distributions of the dependent variables before selecting the appropriate test statistic. We used Pearsons' χ^2^ test to measure associations between categorical sociodemographic characteristics and failure to seek treatment or non-adherence.

Univariate associations for two outcomes (failure to seek treatment or non-adherence to AED) were investigated with logistic regression, adjusted for age. We included variables with p≤0·20 in a multivariable model using a backward elimination process. Variables with p≤0·05 in multivariable analysis were retained in the final model. Attributable fractions were calculated with the final multivariable logistic regression model by a user-written command “punaf” in STATA version 12.^32^, ^33^

### Role of the funding source

The sponsor of the study had no role in the study design, data collection, data analysis, data interpretation, or writing of the report. The corresponding author had full access to all the data in this study and had final responsibility for the decision to submit for publication.

## Results

The epidemiological survey included 232 176 (99%) of 233 881 people living in the KHDSS region during 2008. 699 people with epilepsy were identified, of whom 673 (96%) were interviewed (figure). 392 (58%) participants were younger than 18 years. Many adults (133 [47%]) had no formal education.

**Figure:**
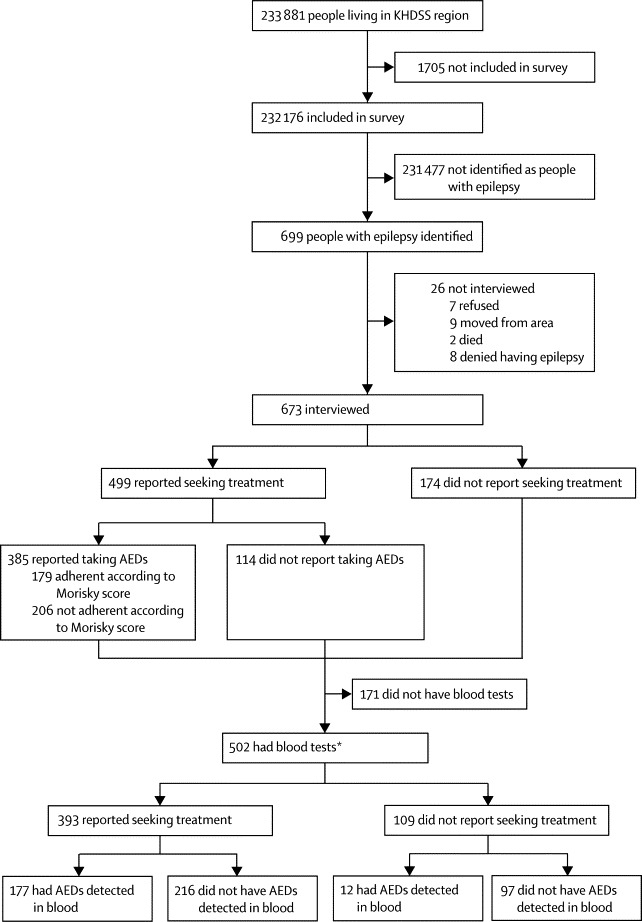
Study profile KHDSS=Kilifi health and demographic surveillance system. AED=antiepileptic drug. *Complete data available for 341 participants: 172 judged to be adherent on the basis of AED detection in blood, 169 did not have AED detected in blood.

Of the 673 people with epilepsy who were interviewed, 499 (74%) reported seeking treatment for epilepsy from a health facility, of whom 395 (79%) used KDH as their primary source of AEDs (appendix p 1). 502 (75%) gave blood samples. People who did not give blood samples were significantly younger, were more likely to have traditional religious (animist) beliefs, were more likely to believe that epilepsy responds to biomedical treatment, were more likely to be married (if adults), less likely to pay for AEDs, had epilepsy for a shorter period, had less frequent seizures, and were less likely to injure themselves during the seizures compared with people who provided samples (appendix pp 2–3). Of the 502 people in whom blood concentrations of AEDs were measured, 393 reported seeking treatment, of whom 117 (40%) were on polytherapy. Phenobarbital was detected in the blood of 151 people with epilepsy, phenytoin in 50, and carbamazepine in 48. Four patients reported taking sodium valproate (which was not measured in blood), but two of these patients were taking one of the other AEDs, and the other two had not collected their drug from KDH (the only source of sodium valproate) within the past 3 months and were classified as non-adherent. The epilepsy treatment gap based on AEDs detected in blood samples was 62·4% (95% CI 58·1–66·6).

We studied 19 variables as potential predictors of people not seeking biomedical treatment (table 1). Of these, 16 had univariate p values of 0·20 or less and were included in the multivariable logistic regression model. From the multivariable analysis, people with epilepsy were less likely to seek treatment if they had traditional animistic religious beliefs, had negative attitudes about biomedical treatment, lived 20 or more km from where they sought health care, reported paying for AEDs, had epilepsy for longer than 10 years, had learning difficulties, and had focal seizures (table 2). Attributable fraction analysis indicated that treatment seeking could improve by 11% (95% CI 1–21) if people with epilepsy thought that biomedical treatment would help, 40% (21–54) if AEDs were free, and 12% (3–21) if the health facilities where people obtained AEDs were less than 20 km from their homes.^32^, ^33^

**Table 1:** Univariate analysis of factors associated with failure to seek biomedical treatment

	**Sought biomedical treatment (n=499)**	**Never sought biomedical treatment (n=174)**	Odds ratio*(95% CI)	**p value**
**Sex**				
Female	239 (48%)	93 (53%)	1·0	..
Male	260 (52%)	81 (47%)	0·81 (0·57–1·14)	0·227
**Religion**				
Christianity	237 (47%)	58 (33%)	1·0	..
Islam	58 (12%)	23 (13%)	1·65 (0·94–2·90)	0·081
Traditional	204 (41%)	93 (53%)	1·83 (1·26–2·68)	0·002
**Education**				
None	231 (46%)	75 (43%)	1·0	..
Primary	227 (45%)	89 (51%)	1·28 (0·89–1·84)	0·178
Secondary	31 (6%)	9 (5%)	0·96 (0·44–2·13)	0·927
Tertiary	10 (2%)	1 (1%)	0·26 (0·03–2·09)	0·207
**Marital status**				
Single	97 (19%)	20 (11%)	1·0	..
Married	313 (63%)	121 (70%)	1·97 (1·16–3·34)	0·012
Separated	9 (2%)	2 (1%)	0·94 (0·18–4·75)	0·940
Divorced	27 (5%)	12 (7%)	2·18 (0·94–5·02)	0·068
Widowed	53 (11%)	19 (11%)	1·65 (0·81–3·39)	0·168
**Occupation**				
Subsistence farmer	271 (54%)	102 (59%)	1·0	..
Trader	111 (22%)	34 (20%)	0·85 (0·54–1·33)	0·473
Casual	41 (8%)	20 (11%)	1·30 (0·73–2·33)	0·372
Other	76 (15%)	18 (10%)	0·60 (0·34–1·06)	0·079
**Socioeconomic status**				
Least poor	110 (22%)	24 (14%)	1·0	..
Less poor	95 (19%)	39 (22%)	0·99 (0·58–1·71)	0·981
Poor	94 (19%)	40 (23%)	1·21 (0·71–2·01)	0·489
Very poor	97 (19%)	35 (20%)	1·15 (0·68–1·97)	0·599
Most poor	103 (21%)	36 (21%)	0·62 (0·35–1·11)	0·108
**Beliefs about causes of epilepsy**†				
Median	8 (5–10)	8 (4–10)	0·90 (0·85–0·95)	0·0002
**Beliefs about biomedical treatment**†				
Median	16 (15–16)	16 (14–16)	0·84 (0·78–0·91)	<0·0001
**Beliefs about cultural treatment**†				
Median	12 (8–15)	12 (7–15)	0·98 (0·94–1·01)	0·243
**Risk and safety concerns about epilepsy**†				
Median	8 (8–8)	8 (7–8)	0·90 (0·79–1·02)	0·109
**Stereotype about epilepsy**†				
Median	8 (6–12)	8 (5–12)	1·00 (0·96–1·04)	0·915
**Stigma scores**‡				
Median	7 (2–13)	6 (2–12)	0·98 (0·96–1·01)	0·131
**Distance to health facility**				
<10 km	177 (35%)	36 (21%)	1·0	..
10–19 km	169 (34%)	65 (37%)	1·82 (1·15–2·89)	0·011
20–30 km	127 (25%)	51 (29%)	1·91 (1·18–3·11)	0·009
>30 km	26 (5%)	22 (13%)	4·02 (2·04–7·89)	0·021
**Payment for AEDs**				
No	195 (39%)	32 (18%)	1·0	..
Yes	304 (61%)	142 (82%)	2·92 (1·91–4·48)	<0·0001
**Learning difficulties**				
No	346 (69%)	146 (84%)	1·0	..
Yes	153 (31%)	28 (16%)	2·23 (1·42–3·50)	0·0005
**Duration of epilepsy**				
<1 year	18 (4%)	26 (15%)	1·0	..
1–5 years	167 (33%)	70 (40%)	1·05 (0·62–1·78)	0·086
6–10 years	121 (24%)	28 (16%)	2·03 (1·32–3·19)	0·001
>10 years	193 (39%)	50 (29%)	7·20 (3·57–14·5)	<0·0001
**Number of seizures in past 3 months**				
None	146 (29%)	63 (36%)	1·0	..
1–3	166 (33%)	54 (31%)	0·73 (0·47–1·12)	0·144
4–6	72 (14%)	22 (13%)	0·72 (0·41–1·23)	0·246
>6	115 (23%)	35 (20%)	0·71 (0·44–1·16)	0·170
**Focal seizures**				
No	157 (31%)	88 (51%)	1·0	..
Yes	342 (69%)	86 (49%)	2·15 (1·51–3·07)	<0·0001
**Injury during seizure**				
No	230 (46%)	102 (59%)	1·0	..
Yes	269 (54%)	72 (41%)	0·50 (0·35–0·72)	0·0002

Data are number of patients (%), median (IQR), odds ratio (95% CI), or p value. AED=antiepileptic drug. For the variables of religion, education, marital status, occupation, socioeconomic status, beliefs and attitudes, and stigma scores, the information pertains to either the person with epilepsy, or if the person with epilepsy was a child or cognitively impaired, to the carer, since the carer made the decision about treatment seeking and giving the AED.

* Adjusted for age.

† Scores of the Kilifi belief and attitude scale were based on a Likert scale (0=not at all, 1=believe a little, 2=totally believe), which were summated for the subscale score; higher scores represent more positive beliefs about biomedical treatment.^26^

‡ Scores of the Kilifi stigma scale for epilepsy were based on a Likert scale (0=not at all, 1=sometimes, 2=always), which were summated; higher scores represent more stigmatisation.^25^

**Table 2:** Multivariable analysis of factors associated with failure to seek biomedical treatment

		Odds ratio*(95% CI)	**p value**
**Predisposing factors**			
Religion			
	Christian	1·0	..
	Islam	1·52 (0·79–2·91)	0·211
	Traditional	1·85 (1·11–2·71)	0·015
Negative beliefs about biomedical treatment		0·86 (0·78–0·95)	0·003
**Health-care system factors**			
Distance to health facility			
	<10 km	1·0	..
	10–19 km	1·56 (0·92–2·62)	0·097
	20–30 km	1·93 (1·11–3·35)	0·019
	>30 km	3·89 (1·77–8·51)	0·0007
**Enabling factors**			
Payment for AEDs			
	No	1·0	..
	Yes	2·99 (1·82–4·92)	<0·0001
**Need-specific and disease-specific factors**			
Learning difficulties			
	No	1·0	..
	Yes	2·30 (1·29–4·11)	0·005
Duration of epilepsy			
	<1 year	1·0	..
	1–5 years	0·72 (0·40–1·30)	0·280
	6–10 years	1·08 (0·63–1·87)	0·764
	>10 years	4·60 (2·07–10·23)	<0·0001
Focal seizures			
	No	1·0	..
	Yes	2·28 (1·50–3·47)	0·0001

AED=antiepileptic drug. For the variables of religion, beliefs, and attitudes about epilepsy, the information pertains to either the person with epilepsy, or if the person with epilepsy was a child or cognitively impaired, to the carer, since the carer made the decision about treatment seeking and giving the AED.

* Adjusted for age.

Of the 499 people who reported seeking biomedical treatment, only 385 (77%) were prescribed AEDs and the person with epilepsy or their carer were able to answer the questions on the Morisky scale. On the basis of Morisky scores, 179 (46%) people with epilepsy were adherent (score of 0) and 206 (54%) were non-adherent (score >0; table 3). Of the 502 who had AEDs measured in blood, 189 (38%) had AEDs detected of whom 72 reported taking AEDs. In the 313 who did not have any AED detected, 60 (19%) reported taking AEDs. 48 (25%) of the 189 people with AEDs detected in their blood had concentrations in the detectable but not optimum range and 141 (75 %) were in the optimum range. Overall, the sensitivity and specificity of reported adherence compared with AEDs detected in the blood were 38·1% (95% CI 31·1–45·4) and 80·8% (76·0–85·0), respectively. The concordance between self-reported adherence and detection in blood for the three prescribed AEDs occurred in 72 (55%) of 132 people with epilepsy. There was little agreement between adherence as measured by blood samples and the Morisky scores.

**Table 3:** Adherence as assessed with the self-reported Morisky scale compared with that as assessed by detection of AEDs in blood samples

	Patients who answered “No”*	**Patients with AED detected in blood who answered “No”**†	**κ**‡
Do you ever forget to take your medication?	112 (29%)	41 (37%)	−0·048
Are you careless at times about taking your medication?	114 (30%)	37 (32%)	0·099
When you feel better, do you sometimes stop taking your medication?	44 (11%)	19 (43%)	−0·004
Sometimes, if you feel worse when you take your medication, do you stop taking it?	29 (8%)	13 (45%)	−0·022

Data are n (%), n, %, or κ. AED=antiepileptic drug.

* Percentages of the 385 people with epilepsy who were prescribed AEDs and they or their carer was able to answer the questions on the Morisky scale.

† Percentages of the participants who had answered “No” to the relevant Morisky question.

‡ κ score of “No” on Morisky scale versus detectable blood concentrations of AEDs.

We studied 25 variables as potential predictors of non-adherence (table 4). 15 were retained in the multivariable logistic regression model. People with epilepsy were less likely to adhere if they had negative attitudes such as feeling that they were rejected or had taken AEDs for more than 5 years (table 5). Attributable fraction analysis indicated that adherence could be improved by 20% (95% CI 7–30) if attitudes could be modified.

**Table 4:** Univariate analysis of factors associated with non-adherence to AEDs

	**Non-adherent people (n=172)**	**Adherent people (n=169)**	Odds ratio*(95% CI)	**p value**
**Sex**				
Female	74 (43%)	82 (49%)	1·0	..
Male	98 (57%)	87 (51%)	1·25 (0·81–1·92)	0·299
**Religion**				
Christianity	85 (49%)	91 (54%)	1·0	
Islam	23 (13%)	18 (11%)	1·38(0·70–2·75)	0·352
Traditional	60 (36%)	64 (37%)	1·13 (0·71–1·79)	0·613
**Education**				
None	70 (41%)	74 (44%)	1·0	..
Primary	85 (49%)	76 (45%)	1·20 (0·76–1·88)	0·433
Secondary	11 (6%)	16 (9%)	0·75 (0·32–1·74)	0·502
Tertiary	6 (3%)	3 (2%)	2·03 (0·48–8·46)	0·336
**Marital status**				
Single	34 (20%)	52 (31%)	1·0	..
Married	108 (63%)	82 (49%)	2·02 (1·20–3·39)	0·008
Separated	1 (1%)	5 (3%)	0·29 (0·03–2·60)	0·267
Divorced	9 (5%)	10 (6%)	1·40 (0·52–3·81)	0·508
Widowed	20 (12%)	20 (12%)	1·53 (0·72–3·26)	0·270
**Occupation of household head**				
Farmer	90 (52%)	87 (51%)	1·0	..
Trader	41 (24%)	36 (21%)	1·13 (0·66–1·93)	0·665
Casual	18 (11%)	17 (10%)	1·03 (0·50–2·13)	0·934
Other	23 (13%)	29 (17%)	0·77 (0·42–1·45)	0·428
**Social economic status**				
Least poor	38 (22%)	50 (30%)	1·0	..
Less poor	36 (21%)	35 (21%)	1·64 (0·77–3·48)	0·196
Poor	36 (21%)	34 (20%)	1·11 (0·55–2·25)	0·774
Very poor	35 (20%)	22 (13%)	1·08 (0·53–2·19)	0·831
Most poor	27 (16%)	28 (17%)	0·79 (0·40–1·55)	0·489
**Availability of family support**				
No	19 (11%)	40 (24%)	1·0	..
Yes	143 (83%)	129 (76%)	1·58 (0·92–2·71)	0·097
Unknown	10 (6%)	0	..	..
**Beliefs about causes of epilepsy**†				
Median	8 (5–10)	8 (6–10)	0·94 (0·87–1·01)	0·113
**Beliefs about biomedical treatment**†				
Median	16 (15–16)	16 (15–16)	0·92 (0·80–1·04)	0·178
**Beliefs about cultural treatment**†				
Median	12 (8–16)	13 (9–16)	0·99 (0·95–1·04)	0·716
**Risk and safety concerns about epilepsy**†				
Median	8 (7–8)	8 (8–8)	0·82 (0·68–0·98)	0·034
**Beliefs about epilepsy**†				
Median	8 (4–11)	10 (6–13)	1·08 (1·03–1·15)	0·002
**Stigma scores**‡				
Median	7 (2–13)	9 (4–15)	0·97 (0·94–1·00)	0·088
**Learning difficulties**				
No	127 (74%)	107 (63%)	1·0	..
Yes	45 (26%)	62 (37%)	0·62 (0·39–0·99)	0·047
**Distance to health facility**				
<10 km	44 (26%)	63 (37%)	1·0	..
10–19 km	61 (35%)	45 (27%)	1·92 (1·11–3·31)	0·019
20–30 km	58 (34%)	49 (29%)	1·70 (0·99–2·94)	0·053
>30 km	9 (5%)	12 (7%)	1·07 (0·41–2·75)	0·893
**Good relation with health-care provider**				
No	19 (11%)	24 (14%)	1·0	..
Yes	153 (89%)	145 (86%)	1·34 (0·70–2·55)	0·374
**Payment for AEDs**				
No	116 (67%)	88 (52%)	1	..
Yes	56 (33%)	81 (48%)	1·97(1·26–3·06)	0·003
**AEDs stored out of sight**				
No	127 (74%)	107 (63%)	1·0	..
Yes	45 (26%)	62 (37%)	1·62 (1·02–2·57)	0·043
**Duration of epilepsy**				
<1 years	4 (2%)	1 (1%)	1·0	..
1–9 years	91 (53%)	87 (51%)	0·26 (0·03–2·41)	0·238
10–20 years	51 (30%)	60 (36%)	0·21 (0·02–1·97)	0·173
>20 years	26 (15%)	21 (12%)	0·30 (0·03–2·88)	0·294
**Focal seizures**				
No	54 (31%)	50 (30%)	1·0	..
Yes	118 (69%)	119 (70%)	0·91 (0·57–1·45)	0·683
**Number of seizures in past 3 months**				
None	46 (27%)	42 (25%)	1·0	..
1–3	68 (40%)	44 (26%)	1·41 (0·80–2·49)	0·230
4–6	28 (16%)	28 (17%)	0·92 (0·47–1·81)	0·811
>6	30 (17%)	55 (33%)	0·50 (0·27–0·93)	0·029
**Duration of AED treatment**				
<1 year	49 (28%)	17 (10%)	1·0	..
1–3 years	52 (30%)	48 (28%)	1·68 (0·83–3·39)	0·147
4–5 years	21 (12%)	21 (12%)	1·80 (1·06–3·04)	0·029
>5 years	50 (29%)	83 (49%)	4·76 (2·47–9·15)	<0·0001
**Number of AEDs**				
Monotherapy	115 (67%)	87 (51%)	1·0	
Polytherapy	57 (33%)	82 (49%)	0·52 (0·34–0·81)	0·004
**Reported side-effects of AEDs**				
No	156 (91%)	157 (93%)	1·0	
Yes	16 (9%)	12 (7%)	1·36 (0·62–2·96)	0·460
**Injury during seizure**				
No	69 (40%)	54 (32%)	1·0	
Yes	103 (60%)	115 (68%)	0·65 (0·41–1·04)	0·071

Data are number of patients (%), median (IQR), odds ratio (95% CI), or p value. The 341 patients included in this table are drawn from the 393 who reported seeking treatment, and had more than 90% data available for the variables examined. For the variables of religion, education, marital status, occupation, socioeconomic status, beliefs and attitudes, and stigma scores, the information pertains to either the person with epilepsy, or if the person with epilepsy was a child or cognitively impaired, to the carer, since the carer made the decision about treatment seeking and giving the AED. AED=antiepileptic drug.

* Adjusted for age.

† Scores of the Kilifi belief and attitude scale were based on a Likert scale (0=not at all, 1=believe a little, 2=totally believe), which were summated for the subscale score; higher scores represent more positive beliefs about biomedical treatment.^26^

‡ Scores of the Kilifi stigma scale for epilepsy were based on a Likert scale (0=not at all, 1=sometimes, 2=always), which were summated; higher scores represent more stigmatisation.^25^

**Table 5:** Multivariable analysis of factors associated with non-adherence to AEDs

	Odds ratio*(95% CI)	**p value**
**Attitudes**		
Negative attitudes about epilepsy	1·10 (1·03–1·18)	0·004
**Duration of AED treatment**		
<1 year	1·0	
1–3 years	1·75 (0·79–3·87)	0·165
4–5 years	1·79 (0·98–3·29)	0·059
>5 years	3·78 (1·79–7·98)	0·0005

AED=antiepileptic drug. For attitudes about epilepsy, the information pertains to either the person with epilepsy, or if the person with epilepsy was a child or cognitively impaired, to the carer, since the carer made the decision about treatment seeking and giving the AED.

* Adjusted for age.

## Discussion

In Kenyans with active convulsive epilepsy who had similar characteristics to people with epilepsy in other parts of Africa,^3^, ^34^ we found that the epilepsy treatment gap was 62% as measured by detection of AEDs in blood samples. This finding represents a reduction from 74% measured during a survey in 2003^10^ and could be attributed to setting up an epilepsy clinic with a continuous supply of AEDs and sensitisation of the community. However, the sensitivity and specificity of self-reporting remained poor. The factors associated with reduced treatment seeking are different to those associated with reduced adherence to AEDs. Modifiable risk factors for failure to seek treatment are negative attitudes about biomedical treatment, payment for AEDs, and distance from health facilities providing AEDs. Adherence to AEDs could be improved by reduction of negative attitudes to epilepsy (panel).

Panel **Research in context**

**Systematic review**

We searched Medline (1966, to May, 2012), ISI Web of Science (1966, to May, 2012), and Cochrane reviews with the search term “epilepsy treatment gap”. Searches were restricted to human studies. We found 281 references, most of which we reviewed in a previous paper^3^ and a recent review.^4^ We reviewed the outstanding 31 references. We found that the epilepsy treatment gap was attributed to cultural beliefs, inadequate skilled manpower, cost of treatment, and unavailability of antiepileptic drugs.

**Interpretation**

This study showed that self-reporting of adherence to antiepileptic drugs (AEDs) might not be reliable in rural Africa, and that risk factors for the failure to seek biomedical treatment for epilepsy and the poor adherence to prescribed treatment are different. Treatment seeking could be improved by modification of attitudes to epilepsy and provision of free AEDs in health facilities near to the residence of the person with epilepsy. Adherence could be improved by changes in attitudes towards epilepsy.

People with epilepsy who held traditional animistic religious beliefs were less likely to seek biomedical treatment than were Christians and Muslims, possibly because of misconceptions and superstitions associated with epilepsy in Kilifi.^35^ People with epilepsy with negative beliefs and attitudes about biomedical treatment of epilepsy were less likely to seek treatment from health facilities, suggesting that modification of patients' beliefs and attitudes is an important step in improvement of treatment seeking.^36^, ^37^

Other factors associated with failure to seek treatment were distance to health facilities and payment for AEDs. The geographical proximity of health services to people's homes is an important factor that affects use of health services, particularly in resource-poor settings where density of biomedical health facilities is often low. The 1997 Kenyan health policy strategic framework states that all households should have access to health services within a 5 km range.^38^ In KHDSS, there is a high density of biomedical health facilities within reach by people with epilepsy, but only KDH has an epilepsy clinic with a continuous supply of AEDs and AEDs other than phenobarbital. The erratic supplies of AEDs in the peripheral clinics (JAC, unpublished) might have reduced the use of these peripheral facilities. People with epilepsy who reported paying for AEDs were less likely to seek treatment than were people who did not pay. The cost of AEDs in peripheral facilities was 10 KES (US$0·13) for a 1-month supply of phenobarbital in 2008, whereas at KDH it was 40 KES ($0·51). This cost of AEDs, although small, is significant when combined with indirect costs of seeking care—eg, transport costs.

Of the epilepsy-specific factors, people with epilepsy who had seizures for more than 10 years might be less likely to seek treatment compared with people with disease of shorter duration because they had learnt to cope with their disorder. People with focal seizures were less likely to seek treatment than were those with generalised seizures, although focal seizures are associated with symptomatic causes and are more likely to be associated with behavioural problems.^39^ Most of the seizures were focal becoming generalised, and being aware at the onset of seizure might have made people less inclined to seek treatment because of the stigma. People with epilepsy and learning difficulties were less likely to seek treatment, perhaps because they were not brought to biomedical facilities by their families or because of their difficulties in understanding the importance of treatment.

Patient self-reporting using reliable and valid questionnaires is thought to be an efficient and cost-effective method to assess adherence, although some patients might underestimate or overestimate their adherence.^40^ We found that the sensitivity and specificity of self-reporting compared with detection of AEDs in blood samples was low, suggesting that self-reporting is not a reliable measure of adherence in people with epilepsy in this community and probably in many countries with low and middle incomes. This finding questions the reliability of data in systematic reviews of the epilepsy treatment gap.^3^, ^4^ Furthermore, we did not find that the Morisky scale was useful for measuring adherence in this population. Although the scale has been used in other studies of epilepsy,^28^, ^29^ it has not been compared with AED detection in blood. There was no significant difference in adherence based on blood samples among the different AEDs (data not shown), despite the different half-lives (phenobarbital, 48–168 h; phenytoin, 7–42 h; carbamazepine, 5–26 h).^30^

Non-adherence was associated with established epilepsy, which is in accord with previous reports that people with epilepsy who had taken AEDs for a longer duration were less likely to adhere.^41^ Non-adherence was also associated with negative beliefs and attitudes about epilepsy as reported previously.^6^

Our study had several limitations. Non-adherence was reported at only one timepoint as opposed to monitoring over time, which might capture variations such as differences in timing of administration, missing doses of AEDs, or taking AEDs just before clinical assessment.^42^ We did not study people with non-convulsive epilepsy since the survey was designed to capture only those with active convulsive epilepsy. Recall and reporting bias of self-reported answers might have occurred, and there were significant differences between people who gave blood samples for measurement of adherence and those who did not. This study was done in an area where we have been studying epilepsy since 2003, although the treatment gap is still 62%. We did not control for quality of care, which might play a part in determining health service use.

In this rural area of Kenya, almost two-thirds of people with epilepsy were not adequately treated and the sensitivity and specificity of self-reported adherence were poor. Modification of beliefs and attitudes and provision of a free and constant supply of AEDs to nearby health facilities could substantially reduce the treatment gap.
