# Supplementary material for: Risk factors associated with the epilepsy treatment gap in Kilifi, Kenya: a cross-sectional study
Source: Lancet Neurol. 2012 Aug;11(8):688–96. doi: 10.1016/S1474-4422(12)70155-2 (PMC3404220; doi:10.1016/S1474-4422(12)70155-2)
Supplement: Supplementary appendix [file mmc1.pdf]

THE LANCET Neurology

Supplementary webappendix

This webappendix formed part of the original submission and has been peer reviewed. We post it as supplied by the authors.

Supplement to: Mbuba CK, Ngugi AK, Fegan G, et al. Risk factors associated with the epilepsy treatment gap in Kilifi, Kenya: a cross-sectional study. *Lancet Neurol* 2012; published online July 6. http://dx.doi.org/10.1016/S1474-4422(12)70155-2.

Figure: Distances to health facility used by people with epilepsy to seek biomedical treatment

Euclidian distance to the health facility where the person with epilepsy (PWE) sought treatment (n=499). Background shading represents quintiles of the distance to Kilifi District Hospital (KDH), the health facility used by 79% of people with epilepsy accessing treatment.

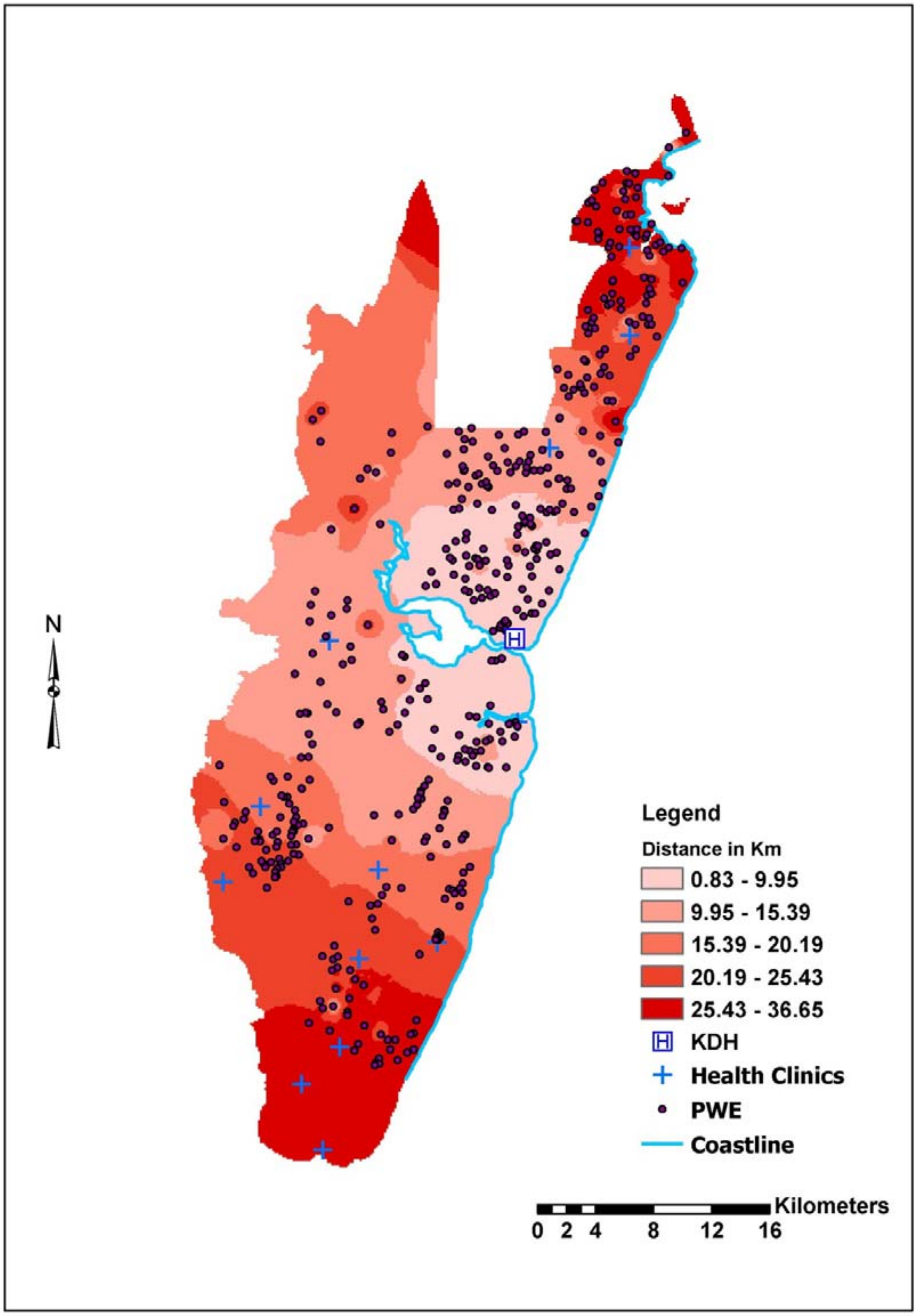

Variable	Blood samples available (n=502)		Blood samples not available (n=171)		P-Value
	n	%	n	%	
**Age:** median(IQR)	502	17.5(11-29)	171	12(6-21)	<0.001
**Sex : f**emale	248	49.4	84	49.1	0.950
**Religion**					
Traditional	204	40.6	93	54.4	
Christian	231	46.0	64	37.4	
Islam	67	13.4	14	8.2	0.005
**Education level:**					
None	223	44.4	83	48.5	
Primary	239	47.6	77	45.0	
Secondary	29	5.8	11	6.4	
Tertiary	11	2.2	0	0	0.213
**Marital status:**					
Single	101	20.1	16	9.4	
Married	305	60.8	129	75.4	
Separated	8	1.6	3	1.8	
Divorced	30	6.0	9	5.2	
Widowed	58	11.6	14	8.2	0.007
**Occupation:**					
Farmer	269	53.6	104	60.8	
Trader	112	22.3	33	19.3	
Casual	48	9.6	13	7.6	
Other	73	14.5	21	12.3	0.432
**Social economic status:**					
Least poor	105	20.9	29	17.0	
Less poor	96	19.1	38	22.2	
Poor	101	20.1	33	19.3	
Very poor	92	18.3	40	23.4	
Most poor	108	21.5	31	18.1	0.401
**Beliefs about causes of epilepsy:** median(IQR)	502	8(6-10)	171	8(4-10)	0.259
**Beliefs about biomedical treatment:** median(IQR)	502	16(15-16)	171	16(14-16)	0.009
**Beliefs about cultural treatment:** median(IQR)	502	12(8-15)	171	12(8-15)	0.798
**Risk and safety concerns beliefs:** median(IQR)	502	8(8-8)	171	8(8-8)	0.644
**Stereotype about PWE :** median(IQR)	502	8(6-12)	171	8(6-12)	0.930
**Stigma score :** Median(IQR)	502	7(2-13)	171	5(2-12)	0.176
**Distance to health facility(Kms)**	**502**	15.7(0.43)	171	16.61(0.66)	0.293
**Paying for AEDs:**	181	36.1	46	26.9	0.029

**Learning difficulties:**	136	27.1	45	26.3	0.843
**Duration of epilepsy**					
<1	27	6.6	17	12.8	
1-3	35	8.6	23	17.39	
3-5	36	8.9	10	7.5	
>5	309	75.9	83	62.4	0.002
**How often have you had seizures in the last 3 months**					
None	142	28.3	67	39.2	
1-3	163	32.5	57	33.3	
4-6	78	15.5	16	9.4	
>6	119	23.7	31	18.1	0.017
**Focal seizures**	170	33.8	58	33.9	0.998
**Injury during seizures: mean (SE)**	502	55.0	171	39.2	<0.001
**No of AEDs:**					
Monotherapy	177	60	43	70.5	
Polytherapy	118	40	18	29.5	0.160
**Duration of medication**					
<1	57	19.3	14	23.0	
1-3	86	29.2	19	31.2	
4-5	36	12.2	5	8.2	
>5	116	39.3	23	37.7	0.767
**Reported side effects of AED**	24	8.1	8	13.1	0.216
**AED stored out of sight**	200	67.8	48	78.7	0.092
**Good relation with provider**	254	86.1	57	93.4	0.116
**Availability of family support**	238	80.7	49	80.3	0.959
**Self-reported non-adherence**	163	55.3	29	47.5	0.271

Supplementary Table: Comparison between those who gave blood samples and those who did not give blood samples.
